# Loneliness and Diurnal Salivary Cortisol in Emerging Adults

**DOI:** 10.3390/ijms19071944

**Published:** 2018-07-03

**Authors:** Julian Chuk Ling Lai, Monique On Yee Leung, Daryl Yu Heng Lee, Yun Wah Lam, Karsten Berning

**Affiliations:** 1 Psychophysiology Laboratory, Department of Social and Behavioural Sciences, City University of Hong Kong, Hong Kong, China; moyleung2-c@my.cityu.edu.hk (M.O.Y.L.); yhdlee2-c@my.cityu.edu.hk (D.Y.H.L.); 2 Department of Chemistry, City University of Hong Kong, Hong Kong, China; yunwlam@cityu.edu.hk (Y.W.L.); karsten.berning@hotmail.de (K.B.)

**Keywords:** loneliness, depression, salivary cortisol, Chinese undergraduates, emerging adults

## Abstract

This study aimed to examine the relationship between trait loneliness and diurnal rhythms of salivary cortisol. Fifty-One Chinese undergraduates provided six saliva samples on a weekday at immediately, 0.5, 3, 6, and 12 h after waking, and at bedtime. Saliva collection times were monitored using electronic devices (MEMS TrackCaps). Participants were also administered a questionnaire consisting of scales measuring, trait loneliness, depression, and demographics. Relationships between loneliness and the cortisol awakening response (CAR), diurnal slope (DS), and area under the curve with respect to ground (AUC_G_) were examined using multiple regression analyses. Results showed that a higher loneliness score was associated with an attenuated CAR, a large AUC_G_, and a steeper DS, with the effects of compliance, waking time, and depression being controlled. As a blunted CAR and a higher diurnal cortisol level have been shown to be associated with poorer health in prior studies, increased attention to the mechanisms translating loneliness into disease endpoints via elevated cortisol is warranted.

## 1. Introduction

Loneliness (or perceived social isolation) [1] has emerged as an important determinant of the physiological processes of stress and increased morbidity in recent research [2,3,4]. As an extensively researched biomarker of health, cortisol has been studied in relation to loneliness in recent years across different age groups (e.g., [5,6]). Dysregulation in the secretion or diurnal rhythm of cortisol has been shown to be detrimental to both physical and mental health [7,8]. There is evidence suggesting that the association between loneliness and increased morbidity is mediated by a potentiation of pro-inflammatory cytokines (e.g., [9,10,11]) possibly induced by chronically elevated cortisol (e.g., [12,13]). According to the glucocorticoid resistance hypothesis [13], an attenuated responsiveness to glucocorticoids (glucocorticoid resistance) is partially attributed to impaired functioning of the glucocorticoid receptor (GR). Glucocorticoid resistance may then lead to elevated cortisol and excessive inflammation having significant implications for a variety of disease, as well as behavioral outcomes.

However, the integration of findings regarding the association between trait loneliness and cortisol has been challenging. For instance, although a positive association between trait loneliness and mean cortisol levels over the course of a day has been reported in college students [14], the same pattern of relationship has not been observed in subsequent studies with similar samples (e.g., [6,15]). With regard to the cortisol awakening response (CAR), the enhanced cortisol awakening response in lonely individuals reported previously [16] has not been replicated in two recent studies [6,17]. Using multilevel modeling, researchers [6] have found that among young adults, higher trait loneliness is associated with a flatter diurnal slope and a slower rate of change in linear decline. However, these findings have not been replicated in a recent study with college students of a similar age [18]. In fact, an increase in loneliness has been found to be associated with a steeper decline in cortisol. The reasons for mixed findings are not immediately apparent but could be attributed to the weak relationship between loneliness and cortisol (*r_s_* < 0.25, [14]), the variation in how frequently cortisol was assessed over the course of a day, the way a particular diurnal component of cortisol was operationalized, the age of participants, or a combination of these factors. The interpretability of the aforementioned findings on loneliness and cortisol is further undermined by the limited number of studies in which saliva sampling times are objectively monitored (e.g., [17]). This is particularly relevant to studies examining cortisol levels in the post-awakening period due to the volatile nature of cortisol levels during this period (e.g., [19]).

In response to the aforementioned gap, we designed the present study to re-examine the relationship between loneliness and salivary cortisol in Chinese young adults, with saliva sampling times monitored objectively using electronic devices (MEMS TrackCaps, AARDEX, Sion, Switzerland). Loneliness has been shown to be associated with higher depressive symptomatology longitudinally [20], and major depression has also been found to be associated with an elevated CAR (e.g., [21,22]), but prior studies rarely controlled the effect of this variable in relation to that of loneliness on cortisol. To address this issue, the relationship between loneliness and cortisol was examined with depression as one of the covariates in the present study. We chose to study a young healthy sample of undergraduates because the challenges inherent to the higher education environment [23] and emerging adulthood [24] create a suitable context for examining how the interplay between a challenging environment and vulnerability factors such as loneliness is associated with cortisol. The concept of emerging adulthood was put forward originally to denote a specific developmental stage between the late teens through the twenties which is distinguished from adolescence and young adulthood by a larger scope of exploration of different life directions [24]. In most industrialized countries, this is the period in which many young people obtain higher education and other professional training that provide the foundations for their occupational achievements in subsequent years of their adult work lives.

The assessment of cortisol levels or diurnal rhythms has been a major challenge for researchers because of the daily rhythm of cortisol. The circadian rhythm of cortisol is characterized by a substantial rise within the first hour after waking with a peak at about 30–45 min post-waking (i.e., CAR) [25] and a gradual decline over the course of the day until the nadir is reached around midnight; the level rises gradually during nocturnal sleep until waking the next morning [26,27]. Recent research has revealed that the circadian rhythm of cortisol is under the control of a SCN-PVN (suprachiasmatic nucleus-periventricular nucleus) and a SCN-adrenocortical pathway [28]. The former controls the release of cortisol from the adrenal cortex via CRH/ACTH (corticotropin-releasing hormone/adrenocorticotropic hormone) and the latter controls the sensitivity of the adrenal cortex to ACTH.

To accurately assess the changes of cortisol levels or rhythms in relation to various psychosocial factors, a number of indices derived from specific components of the diurnal cycle of cortisol have been adopted in prior research. These include (1) the CAR, which captures the increase in cortisol output from immediately to 30 to 45 min after waking and is operationalized as the difference between the waking and 30-min post-waking level (e.g., [25]); (2) diurnal slope (DS), operationalized as the slope of cortisol from the waking or peak level to a level that approximates the nadir (e.g., [29]); and (3) area under the curve with respect to ground (AUC_G_) captures the overall secretion of cortisol over a specific time period (e.g., [30]). These three indices were used in the present study to provide an accurate characterization of the cortisol profiles associated with trait loneliness.

## 2. Results

Among the 51 participants, twenty-eight failed to self-collect saliva samples at one or more scheduled sampling times. A dummy variable was then created to examine whether non-compliance would affect the results. Missing cortisol data (24 out of 306) due to nondetectable low cortisol concentrations and the absence of MEMS times were imputed with the expectation-maximization method (IBM SPSS 24). As in prior publications by the principal author (e.g., [31]), cortisol data were then normalized by winsorizing to 0.2 nmol/L at the low end for subsequent analyses. Three commonly used indices of the diurnal rhythm of cortisol were computed. The CAR was computed by the percentage of increase in cortisol from waking to 30 min after waking. The DS was operationalized as the linear rate of change in cortisol from waking to bedtime: (level at bedtime–level at waking)/the average length of 16 h). AUC_G_ was computed according to the formula proposed previously [30]. To examine whether a significant CAR or diurnal slope in cortisol was demonstrated in the present sample or not, one-way ANOVAs with repeated measures were conducted. The results clearly showed that the cortisol level at 30 min post-awakening was significantly higher than the waking level: *F*(1, 50) = 72.613, *p* < 0.001. In addition, a significant decline of the cortisol level from waking to bedtime was also observed: *F*(1, 50) = 56.889, *p* < 0.001.

Mean diurnal cortisol levels of the participants at each sampling time point over the course of a day and descriptive statistics are shown in Table 1. Zero-order correlations presented in Table 2 show that higher loneliness was associated with higher depression. Higher scores in either loneliness or depression were associated with an attenuated CAR, a steeper DS, and a larger AUC_G_.

The association between loneliness and each of the three cortisol indices was further examined using multiple regression analyses with the effects of gender, age, waking time, compliance, and depression being controlled. In particular, the method of hierarchical entry of predictor variables was used. Gender and age were entered in the first step, which was followed by waking time, compliance, and depression in separate steps. Loneliness was entered in the last step. The results in Table 3 illustrate that higher loneliness scores were associated with a larger AUC_G_, an attenuated CAR, and a steeper DS. The beta values refer to standardized regression coefficients denoting how much a cortisol parameter such as CAR changes in standardized units such as standard deviation when loneliness increases or decreases by one standard deviation; the t values indicate whether the linear relationship between a cortisol parameter and loneliness is significant or not, i.e., whether the slope of the regression line is significantly different from zero or not. As can be seen in Table 3, an increase in the loneliness score by one standard deviation was associated with a decrease of 0.694 of a standard deviation in CAR. The *t* value with a *p* value smaller than 0.05 suggests that the linear association between loneliness and CAR was statistically significant. 

To examine whether steeper diurnal slopes in lonely participants were attributed to higher waking levels of cortisol or lower bedtime levels, the 51 participants were divided into two groups of lonely vs. non-lonely participants at the mean loneliness score (15.76). *t*-Tests were run to test the difference in the mean level of cortisol between the lonely and the non-lonely participants at each of the two sampling times. The results summarized in Table 4 show that significant differences in cortisol concentration were found at waking but not at bedtime. This clearly shows that steeper diurnal slopes in lonely participants were primarily due to higher cortisol levels at waking.

## 3. Discussion

Using electronic devices to monitor sampling times objectively, we have shown that higher loneliness scores were associated with increased cortisol secretion and an attenuated CAR in emerging adults. However, lonely participants also exhibited a steeper decline of cortisol over the course of a day. Although elevation in diurnal cortisol [32] and attenuation in the CAR [33] have been reported to be associated with poorer health status, the implication of a negative association between loneliness and DS is less clear. As mentioned earlier, DS has been found to be either positively [6] or negatively [18] correlated with loneliness. Moreover, the diurnal slopes in these two studies were determined by the nadir because loneliness had no significant association with either the wake-up level of cortisol or CAR. However, differences in diurnal slopes between lonely and non-lonely participants in the present study were primarily determined by the waking level. This implies that the diurnal rhythm of cortisol of lonely participants in the present study is different from that of lonely participants in the two prior studies [6,18].

The reason for the aforementioned discrepancy is not immediately apparent but could be attributed to the use of different measures of loneliness. In particular, loneliness was measured by an adapted three-item version of the Revised UCLA (University of California, Los Angeles) Loneliness Scale [6] or the UCLA Loneliness Scale Version 3 [18]. Although these two measures of trait loneliness should be similar to the one used in the present study because they are all adapted versions of the UCLA Loneliness Scale, this cannot be taken to imply that they measure the same aspects of the phenotype of the trait loneliness. In close connection to this, the findings of a recent genome-wide study [34] suggest that loneliness is only modestly heritable and a trait with polygenic architecture. Moreover, it was pointed out in the same study that loneliness is genetically correlated with other personality and psychiatric traits. Taken together, these findings imply that the phenotype of the trait loneliness is more heterogeneous than has been thought of. This may explain the strong association between loneliness and the waking level of cortisol in the present study, and the mixed findings reviewed earlier.

Despite the significance of present findings, their impact is moderated by a number of issues that might not have been addressed adequately. The relatively small sample size (*N* = 51) may limit the generality of the present findings. It has been pointed out that estimates of CAR based on cortisol data from one single day may have reduced reliability [35]. In addition, our study is not able to illuminate the physiological mechanisms that may translate loneliness into ill health. Although lonely participants produce more pro-inflammatory cytokines in response to acute laboratory stressors (e.g., [10]), how loneliness promotes pro-inflammatory cytokines via prolonged activation of the HPA axis in the long run is not completely understood. It has been suggested that sensitization of the inflammatory response to repeated exposure to stressors may contribute to the long-term development of diseases in overly stressed or vulnerable individuals [36]. However, more research is needed before the role that cortisol plays in this process is better understood. Further research is warranted to test the glucocorticoid resistance hypothesis with vigorous designs.

## 4. Materials and Methods

### 4.1. Participants

Participants in the study (*N* = 51, 28 females) were all ethnic Chinese undergraduates studying at a university in Hong Kong. Ages of participants ranged from 18 years to 29 years (Mean = 20.90 years *SD* = 1.88), and the majority of them (62.7%) were in their second year of study. The participants had no known diagnosis of psychiatric disorders and cardiovascular diseases, were not currently on medications that would potentially affect cortisol levels, and did not smoke habitually. No female participants used oral contraceptives. They were recruited from students in a class of introductory psychology that were willing to take part in the study voluntarily. Course credits and cinema vouchers were given in return for their participation. Conduction of the study was approved by the human subject ethics committee of the Research Grants and Contracts Office of the City University of Hong Kong. Informed consent was obtained from all participants of the study.

### 4.2. Procedure

Participants were invited to a briefing session during which they were given a detailed description of the saliva sampling procedure, and a study package containing written instructions, questionnaires, saliva sampling tubes (Salivette), and an electronic device (MEMS TrackCaps, AARDEX, Sion, Switzerland) used to monitor the timing of saliva sampling. Participants were asked to collect by themselves six saliva samples on a weekday at immediately, 0.5, 3, 6, and 12 h after waking, and at bedtime. The saliva sampling swabs were removed from the original Salivette tubes by the experimenter and put into the vials, which were covered at the top by MEMS TrackCaps. Participants were instructed to open the vial and take out one swab at each designated sampling time. They were told to gently chew and roll the swab around the mouth for 2 min until the swab was saturated with saliva, and then put the wet swab in a Salivette tube. Exercise, smoking, brushing teeth, eating, and taking beverages containing alcohol or caffeine were not allowed 1 h before saliva collection. The importance of exact timing for saliva collection was stressed, and participants were also informed about how the monitoring devices could record their saliva collection times. A window of 5 min was adopted for the morning saliva samples and a 30-min one for the subsequent samples. At the end of the briefing session, participants filled out a questionnaire consisting of scales measuring trait loneliness, depression, and major demographic characteristics. Participants were told to store their saliva samples in their home freezers until returning them to the experimenter. The returned samples were then stored in the laboratory at −20 °C until thawed for biochemical analysis.

### 4.3. Measures

Trait Loneliness was measured with a Chinese version of the eight-item Loneliness scale [37] adapted by using backward translation. The scale consists of six items phrased in the lonely direction (e.g., I lack companionship) and two phrased in the non-lonely direction (e.g., I can find companionship when I want it). To complete the scale, participants indicated how they experienced the feelings depicted in each of the eight items in general using a four-point scale with 1 = never, and 4 = always. A trait loneliness score was computed by adding ratings of the six lonely items and reversed ratings of the two non-lonely items. The mean score and Cronbach’s α of trait loneliness were 15.76 (*SD* = 4.80), and 0.86, respectively, in the present sample.

Depression was measured by a Chinese version of the Center for Epidemiological Studies Depression Scale (CES-D) [38]. The scale consists of 20 items with 16 phrased in a negative or depressed direction (e.g., “I felt depressed”) and four in a positive direction (e.g., “I was happy”). Prior studies have demonstrated the reliability and validity of the scale when it was applied to Chinese samples [38,39]. Participants were asked to indicate the number of days on which they experienced depressive symptoms in the last week using a four-point scale: 1 = none, 2 = one or two days a week, 3 = three or four days per week, and 4 = five days or more per week. A depression score was computed by adding ratings of the 16 depressive items and reversed ratings of the four positive items. The mean and Cronbach’s α of the CES-D in the present sample were equal to 37.04 (*SD* = 8.01) and 0.87, respectively.

### 4.4. Cortisol Assays

Cortisol concentrations were analyzed using an enzyme-linked immunosorbent assay (Enzo Life Sciences, Inc., Farmingdale, NY, USA) similar to those used in prior studies with Hong Kong Chinese (e.g., [31,40]). Assays were conducted at the Laboratory of the Chemistry Department at the City University of Hong Kong. Saliva samples were thawed and centrifuged at 3500 rpm for 10 min at room temperature. Clear supernatant was used for analysis. The sensitivity of the assays was 0.2 nmol/L. The Intra- and inter-assay coefficients of variation were lower than 12%, which is comparable to those of similar assays used in prior studies with Hong Kong Chinese (e.g., [31,40]).

## Figures and Tables

**Table 1:** Means (SEMs) of Sample Characteristics and Cortisol Indices.

Variables	M (SEM)	Range
Age	20.90 (0.27)	18–29
Loneliness	15.76 (0.67)	8–29
Depression	37.04 (1.12)	21–61
Waking time	09:28 (13 min)	07:00–13:17
Cortisol level: waking	7.91 (0.89)	0.43–23.65
Cortisol level: 30 min post-waking	12.78 (0.97)	1.61–26.00
Cortisol level: 3 h post-waking	4.57 (0.59)	0.73–21.08
Cortisol level: 6 h post-waking	3.03 (0.45)	0.20–13.39
Cortisol level: 12 h post-waking	2.06 (0.34)	0.20–15.42
Cortisol level: bedtime	1.80 (0.33)	0.20–15.00
Cortisol indices		
CAR	125.15 (17.47)	−44.46–528.81
Diurnal slope	−0.38 (0.05)	−1.47–0.06
AUC_G_	61.30 (6.57)	11.62–257.32

*N* = 51; M = mean, SEM = standard error of the mean, CAR = cortisol awakening response, AUC_G_ = area under the curve with respect to ground; cortisol concentrations in nmol/L.

**Table 2:** Pearson correlations for loneliness, depression, mean cortisol indices, and covariates.

Variables	2	3	4	5	6	7	8
1. Gender	0.435 **	0.184	0.030	−0.010	−0.060	0.031	0.049
2. Age		0.067	−0.024	−0.184	0.010	0.046	−0.041
3. Waking time			−0.080	−0.013	−0.105	−0.060	0.089
4. Loneliness				0.586 **	−0.611 **	−0.647 **	0.577 **
5. Depression					−0.304 *	−0.481 **	0.371 **
6. CAR						0.629 **	−0.386 **
7. Diurnal slope							−0.521 **
8. AUC_G_							

* *p* < 0.05; ** *p* < 0.01; CAR = cortisol awakening response, AUC_G_ = area under the curve with respect to ground; gender: male = 0, female = 1.

**Table 3:** Linear regression model predicting the three cortisol indices.

Predictor Variables	AUC_G_			CAR			Diurnal Slope		
	β	*t*	*p*	β	*t*	*p*	β	*t*	*p*
Gender	0.037	0.269	0.789	−0.020	−0.154	0.879	0.081	0.636	0.528
Age	−0.064	−0.458	0.649	0.025	0.182	0.856	−0.027	−0.205	0.839
Waking time	0.121	0.975	0.335	−0.159	−1.328	0.191	−0.123	−1.069	0.291
Compliance	0.110	0.840	0.405	0.036	0.282	0.780	0.026	0.216	0.830
Depression	0.054	0.353	0.726	0.104	0.700	0.488	−0.146	−1.020	0.314
Loneliness	0.521	3.265	0.002	−0.694	−4.495	0.000	−0.582	−3.929	0.000

AUC_G_ = area under the curve with respect to ground, CAR = cortisol awakening response; gender: male = 0, female = 1; compliance: non-compliant participants = 0 (*n* = 28), compliant participants = 1 (*n* = 23).

**Table 4:** Means (*SEMs*) of Cortisol Concentrations (nmol/L) in Lonely vs. Non-Lonely Participants at Waking and Bedtime. *** *p* < 0.001.

Sampling Times	Non-Lonely Participants (*n* = 27)	Lonely Participants (*n* = 24)	*t*
Waking	3.73 (0.39)	12.61 (1.28)	−6.961 ***
Bedtime	1.36 (0.23)	2.30 (0.64)	−1.449
